# American Dermatological Association

**Published:** 1876-07

**Authors:** 


### American Dermatological Association.

We have received the following to which we take pleasure in giving publicity.

DEAR SIR:—At an informal meeting of the undersigned, held in Philadelphia, at the rooms of the Section of Practical Medicine, of the American Medical Association, Wednesday June 7th, 1876, after the election of a Chairman and Secretary, pro-tem: it was

*Resolved* To call upon such American Physicians as had evinced a special interest in Dermatology to unite in forming an American Dermatological Association. *Resolved*, That the meeting for organization be held in the University of Pennsylvania, on Wednesday September 6th, 1876, at 6 P. M., or immediately after the close of the meeting of the Section of Dermatology and Syphilology, of the International Medical Congress, on that day.

It is sincerely desired that you will be present and aid in the organization.

Please signify your pleasure to the Secretary at the earliest opportunity, and oblige, Very Truly Yours,

L. D. BULKLEY, Secretary, pro tem,
1 East 33d St., New York.
EDWARD WIGGLESWORTH Jr., Chairman,
Boston, Mass.
LOUIS A. DUHRING, Philadelphia, Pa.
LUNSFORD P. YANDELL, Louisville, Ky.
GEORGE HENRY FOX, New York.
J. E. ATKINSON, Baltimore, Md.

